# Microglia in the aging brain: relevance to neurodegeneration

**DOI:** 10.1186/1750-1326-5-12

**Published:** 2010-03-24

**Authors:** Xiao-Guang Luo, Jian-Qing Ding, Sheng-Di Chen

**Affiliations:** 1 Department of Neurology and Institute of Neurology, Ruijin Hospital, Shanghai Jiao Tong University School of Medicine, Shanghai, PR China; 2 Department of Neurology and Institute of Neurology, First Affiliated Hospital of China Medical University, Shenyang, PR China

## Abstract

Microglia cells are the brain counterpart of macrophages and function as the first defense in the brain. Although they are neuroprotective in the young brain, microglia cells may be primed to react abnormally to stimuli in the aged brain and to become neurotoxic and destructive during neurodegeneration. Aging-induced immune senescence occurs in the brain as age-associated microglia senescence, which renders microglia to function abnormally and may eventually promote neurodegeneration. Microglia senescence is manifested by both morphological changes and alterations in immunophenotypic expression and inflammatory profile. These changes are likely caused by microinvironmental factors, but intrinsic factors cannot yet be completely excluded. Microglia senescence appears to underlie the switching of microglia from neuroprotective in the young brain to neurotoxic in the aged brain. The hypothesis of microglia senescence during aging offers a novel perspective on their roles in aging-related neurodegeneration. In Parkinson's disease and Alzheimer's disease, over-activation of microglia may play an active role in the pathogenesis because microglia senescence primes them to be neurotoxic during the development of the diseases.

## Introduction

Microglia are the representative of immune cells in the relatively immune-privileged central nervous system (CNS) and account for 10% of the total glial cell population in the brain. The initial investigation of microglia came from Rio-Hortega early in the last century, who described microglia as a unique cell type in the CNS with an elongated soma bearing processes extending from both poles of the cell. When severe brain injury happens, microglia cells change their morphology dramatically, migrate to the lesion sites, and proliferate. Proliferated microglia cells phagocytose dying cells and other debris and/or release cytokines to maintain the microenvironment homeostasis and support injured neurons, and thus are beneficial for the neuronal survival. However, in the past decades mounting evidence has also implicated neurotoxic roles of microglia when over-activated in severe injury or neurodegenerative diseases. Hypotheses trying to explain this double-edged feature of microglia have been proposed. Here, we review the senescence-related changes of microglia and focus on their relevance to neurodegeneration.

## Immunosenescence of macrophage lineage

Like macrophages in the periphery, microglia belong to macrophage lineage and are the first and main form of active immune defense in the CNS. Aging of the immune system (immunosenescence) describes a state of profound age-associated changes in the immune system, which contributes to the increased susceptibility to infection of the elderly[1,2]. Although the compromise of adaptive immunity mediated by T and B lymphocytes has gained a significant amount of attention [3-5], the innate system also experiences significant changes with advanced age[2]. As the bridge between adaptive and innate immune system, the aging-associated deterioration of macrophage lineage is of significance. Although not universally consistent, accumulating evidence has demonstrated alterations in macrophage functions in advanced age. In rodents, the cytokine production (interleukin 1 (IL-1), tumor necrosis factor (TNF-α)) [6-9] as well as oxidative radicals and iNOS expression[9] were reported to decline in macrophages with aging. However, most literature suggests elevated circulating levels of proinflammatory cytokines[10], which are believed to be produced mainly by macrophages during aging. Apparently contradictory results are also seen regarding receptor expression in macrophages. Renshaw et al. reported a reduced Toll-like receptor (TLR) expression in macrophages with advanced age[11], while Boehmer et al [12]. reported that TLR expression was not affected by age. As macrophages in the periphery, it is reasonable to believe that microglial alterations play crucial roles in increased inflammation in the CNS during aging and in neurodegeneration.

## Microglia activation in the aged brain

It has been proposed that aberrant inflammatory responses play a role in the etiology of several neurodegenerative diseases, such as Parkinson's disease (PD) or Alzheimer's disease (AD)[13,14], in which aging is the most important risk factor. Many studies have demonstrated coexisting neuro-inflammation and neurodegeneration[15,16]. The CNS is relatively immune-privileged, in which the activity of adaptive immunity mediated by lymphocytes is scarce[17]. On the other hand, major histocompatibility complex (MHC) antigen, T- and B-lymphocyte markers, and other immune-cell antigens can be detected on microglia. Thus, microglia has been considered to represent the brain's internal immune system. It is speculated that any changes in microglial activities during aging are key components in influencing the pathogenesis of neurodegeneration.

Microglia normally keep in a quiescent state when not challenged, and they are thus called resting microglia, with ramified morphology and weak expression of function-associated antigens. When triggered by appropriate stimulation, microglia rapidly transformed from a resting state to an activated state with deramified shape and enhanced antigen presentation, which has been graded to a series of stages[18]. As an active sensor and monitor in the brain, microglia are neuroprotective. However, uncontrolled microglia response may be dangerous to the survival of injured neurons or even cause damage to healthy neurons that are afflicted by excessive inflammation. Microglia activation is normally controlled under physiological conditions of the CNS through neuron-glia contact, CD200R-CD200 pathway[19], neuronal electrical activity[20], and/or some soluble neurotransmitters[21]. Despite this microglia-quiescence mechanisms, increased microglia activation in the healthy aged brain has been reported in diverse mammalian species, such human[22], monkey[23,24] and rat[25]. During normal aging of the monkey brain, microglial expression of MHC class II increases with age[23], and the phagocytic activity of microglia increases age-dependently, leading to electron dense inclusions that have the appearance of myelin in microglia[24]. Immunohistochemical studies by using activated microglia marker OX-6 staining also found microglial activation with age-related changes[25]. Additionally, increased pro-inflammatory cytokines and decreased anti-inflammatory cytokines have been demonstrated from lipopolysaccharide (LPS)-activated microglia explanted from aged mice[26], suggesting the abnormal immune state of microglia in the aged brain. All of the above evidence suggests that the inflammatory state of microglia in the aged brain primes them it to be over-responsive to small stimuli that are otherwise well controlled in the young brain. Eventually, the activation of microglia in the aged brain loses control. It is still uncertain what triggers the microglial activation in the healthy aged brain; whether it is caused by degenerating neurons or by abnormal protein aggregation found in clinically normal elders is still unknown[22].

## Microglia activation in the CNS: more blessing than curse?

In the past decade there have been discussions on whether microglia activation is beneficial or detrimental to neurons. Most of the debate has arisen from in vitro studies [27-30], which usually give ambiguous effects of microglia on cultured neurons. These studies indicate that microglia are capable of being both neurotrophic and neurotoxic, depending on the specific stimulus, the severity of injury, and the environment [31-34]. However, exactly under what scenario activated microglia are neuroprotective or neurotoxic and what promotes transformation between the two remains unknown. Microglia are distributed ubiquitously throughout the brain and function as resident macrophages and antigen-presenting cells in the CNS[35,36]. They play vital roles in supporting and maintaining neuronal function, health, homeostasis, and survival in both normal and pathological microenvironment by phagocytosis of potentially deleterious debris and secretion of neurotrophic factors to promote tissue repair. By using in vivo two-photon imaging techniques, Nimmerjahn et al[37]. found that in the healthy, intact brain microglia are actually highly active and survey their microenvironment continually with extremely motile processes and protrusions. The same study also suggests that activated microglia exert a neuroprotective role by shielding the injured sites and phagocytosing damaged tissue. Since microdamages may happen frequently throughout the CNS due to microischemic events or accumulated metabolic products, or even degenerating neurons, it is conceivable that microglia are constantly activated and, in most cases, respond to these microdamages in a neurotrophic fashion [37].

As the sentinel and essential cells of the CNS, microglia are not supposed to be harmful to the neuron. However, if the microglia activation in the brain oversteps the threshold of tolerability, it might contribute to pathology rather than have a sentinel or defensive role[38]. This is probably why microglia activation is notorious for being harmful in the brain. Even though concrete and valid proof of neuroprotection of the activated microglia is lacking, there are studies that provide strong evidence demonstrating the neurotrophic work of activated microglia. In axotomy of the optic nerve of amphibians, a rapid microglia response is exhibited with efficient clearance of myelin debris (which contains inhibiting molecules of axon growth) and finally successful axonal regeneration[39]. Activated microglia can also clear glutamate without evoking inflammatory mediators after traumatic injury and thus reduce neurotoxicity[40]. Grafting of cultured microglial cells into the lesioned spinal cord of adult rats enhances neurite outgrowth[41]. Microglia have been demonstrated to protect neurons against ischemia either by synthesis of tumor necrosis factor[42] or by engulfment of harmful invading neutrophil granulocytes[43]. The protective roles of activated microglia have also been extensively discussed in multiple sclerosis[44]. All of the above studies provide strong evidence that activated microglial cells help injured neurons recover and that microglia is strongly indicated as beneficial to neuron survival. Therefore, activation of microglia is generally more beneficial than detrimental if the microglia is working normally. However, under conditions when microglia are deregulated, their over-activation could be neurotoxic, which could eventually contribute to neurodegeneration.

## Distinct microglia in the aged brain: is it due to so-called microglia senescence?

In contrast to acute CNS injuries, neurodegeneration as in AD and PD is a chronic process that may take decades to develop. In this slowly progressive procedure, activated microglia has been demonstrated to play important detrimental roles [45-47]. This is opposite to the physiological neuroprotective function of microglia in the young brain. The destructive roles of the activated microglia in the aged neurodegenerative brain may result from age-associated microglia senescence, which is similar to immunosenescence of macrophages, the peripheral counterpart of microglia. Microglia senescence renders microglia to function abnormally, to fail to respond correctly to stimuli[48,49] and, eventually, to promote neurodegeneration. The most prominent and also the first identified feature of microglia senescence is the morphological alteration described as "dystrophy."[50] Characteristics of "dystrophic" microglia that were observed in the aged brain include deramification (loss of finely branched cytoplasmic processes), cytoplasmic beading/spheroid formation, shortened and twisted cytoplasmic processes, and instances of partial or complete cytoplasmic fragmentation[50]. Such dystrophic microglia were prevalent and extensively distributed in the brain of older human subjects[50,51], whereas normal ramified microglial morphology with only rare instances of dystrophic microglia are seen in the young brain[49] These observations provide initial evidence of the age-associated changes in microglia in the healthy elderly brain.

Apart from the dystrophic morphology, telomere shortening has also been demonstrated in the aged brain. Telomeres, the physical ends of eukaryotic chromosomes, continually shorten with age and divide due to the inability of DNA polymerase to completely replicate linear DNA molecules. When cells eventually exhaust their replicated potential, they enter replicative senescence, leading to the substantial changes in cell function and gene expression. Previous studies have shown that telomere shortening, which is a sign of cell senescence, occurs in rat microglia overtime in vitro[52], as well as in vivo in rat cerebellum and cerebral cortex with age[53]. Because microglia are the major cell type that are capable of dividing to an appreciable extent in the CNS, the reduction in brain telomere length may be caused by telomere shortening in microglia. More direct evidence in support of telomere shortening in microglia in the aged brain came from studies of Flanary et al. who reported that microglial cells exhibit significant telomere shortening and reduction of telomerase activity during normal aging in rats[54]. Collectively, microglia in the aged brain is distinct from that in the young brain both in morphology and in telomere length, which indicate senescence. However, the senescenced microglia are not universal in the aged brain since scattered dystrophic microglia are usually found alongside normal ramified microglial cells, suggesting that only a subset of microglia become dystrophic, and not all of them are of the same age and functional state[55]. It is possible that the number of this subset increases with aging and ultimately outnumbers the neuroprotective normal-functional microglial cells. These age-associated changes may underlie the alterations of microglial function and their distinct responses to injury.

## Distinct pattern of microglia response to injury in the aged brain

Microglia senescence is also manifested by their functional alterations, such as altered inflammatory profile[26,56], increased immunophenotypic expression[23], and the switch from neuroprotective in the young brain to neurotoxic in the aged brain when activated[48]. Microglia response to injury in the aged brain is distinct when compared to that in the young adult brain regarding the timing of microglia proliferation and presentation after the injury. For example, Conde et al. reported that microglia proliferation in the aged rat brain was significantly higher than in the young rat brain 4 days after axotomy of the facial nerve[49]. Significant differences in the activated microglia on the lesioned side were also observed. In an intracerebral hemorrhage model of senescence-accelerated prone mice (SAMP8) and senescence-accelerated resistant mice (SAMR1), brain neutrophils and reactive astrocytes do not differ in number or distribution between SAMP8 and SAMR1 mice, but activated amoeboid microglia are distributed more abundantly around and inside the hemorrhagic lesions in old SAMP8 mice than in young SAMP8 mice[57]. The improvement of neurological deficits in old SAMP8 was also delayed, indicating the role of the distinct microglia response in the delayed recovery. In a study of intracerebral hemorrhage in young and aged rats, it was observed that microglia activation was significantly higher in young rats than in aged ones when examined three days after hemorrhage [58]. The distinct pattern of microglia response to injury in the aged brain has also been recorded in the 1-methyl-4-phenyl-1,2,3,6-tetrahydropyridine (MPTP)-induced neurotoxicity[59], the model of controlled cortical impact (CCI)[60], cortical stab injury[61], and transient retinal ischemia[62]. However, not all studies reach consistent conclusions. For example, Hurley and Coleman[63] did not find any age-related difference in microglia response in either control or axotomized facial nuclei. These inconsistent findings may be attributed to the diversity of injury models, animal species, methods regarding counting, and the definition of microglia activation. Taken together, it appears that microglia in the aged brain respond to stimuli no less than that in the young brain. The problem seems to be the changes of response patterns and uncontrolled activation, which are the major characters of microglia senescence.

Recent studies give more support to the hypothesis of microglia senescence in the aged brain, which offers a novel perspective on aging-related neurodegeneration by looking at the neurodegenerative process from an alternative point of view. It appears that slow progressive neurodegeneration and associated neuronal cell death may result from impaired microglial cell function. This hypothesis provides a potential therapeutic target of improving microglia function by delaying microglia senescence. However, before this proposal can be fully accepted, there are still some key questions that need to be answered. A critical, fundamental question is what is the difference between "the activated microglia" and "the aged microglia". Even though Streit et al[64] gave a suggestion in the difference between the two, describing that "hypertrophy and retraction and thickening of process" occurs in activation while "deramification, shortening and twisting of processes, cytoplasmic fragmentation" are features of aging, so far no definite biomarker or morphology characters can distinguish the two with certainty. The best way to indicate senescence versus activation is to determine the dynamic activities of microglia and their response to injury and whether they will ultimately return to ramified resting state after stimuli and/or whether the changes occur in a physiological environment free of stimuli. There are still many other questions that are hard to answer so far. Is the activating state of microglia in the aging brain co-existing with or secondary to microglia dystrophy? As microglia are physiologically multi-functional, which specific function of microglia is primarily affected by microglia dystrophy? How is it affected and what is the direct consequence of the affected function? Is the deterioration of a specific microglia function more related to neurodegeneration than some other function? Clearly, much more research is needed to answer these questions.

## What factors cause microglia dysfunction in aging brain?

An important question is whether there are any other factors in addition to aging itself that could promote or delay microglia dysfunction in the aged brain. Are the age-related alterations in microglia function and morphology resulting from intrinsic or extrinsic factors? Does microglia over-activation and malfunction in the aged brain come from their failure to respond correctly to their microenvironment, or from an overloaded toxic microenvironment? It has been demonstrated that although the aged rodent and human myeloid dendritic cells are poorly immunogenic, fully functional myeloid dendritic cells can be generated in vitro from blood monocytes from aged donors[9,65,66], suggesting that there is no age-associated intrinsic defect in this lineage and that the age-related changes in macrophage function may be reversible with the proper environment and stimulation. Isolated microglia from aged brains exhibit decreased process complexity, altered granularity, and increased basal cytokines as compared to those from the young brain, suggesting an elevated inflammatory state in the aged microglia[56]. However, after being stimulated with LPS, the fold-over-basal LPS response remains constant across ages, indicating a comparable inflammatory response machinery in aging microglia to that of young ones[56]. These observations suggest that microglia dysfunction in the aged brain might be more related to the extrinsic events than intrinsic ones.

The notion that extrinsic rather than intrinsic factors are the major determinants for microglia's function is also supported by the regional differences in microglia. It has been shown that in different regions of the brain, microglia activation shows differential phenotypes. Ramified activated microglia are mainly associated with periventricular region, whereas innate, amoeboid phagocytic phenotype of microglia is seen within the deep subcortical region[67]. These observations further suggest that different stimuli or environments induce different morphology and functions of activated microglia.

Extrinsic factors might include neurotransmitter, oxidative stress, neuroendocrine factors[10,68], hormones, and pathological factors such as abnormal protein aggregation[52] that exists in the microenvironment where microglia live. For example, amyloid has been shown to accelerate microglia senescence[52]. Thus, it is conceivable that targeting the factors influencing microglia function in the aged environment might restore at least part of the neuroprotective function of microglia in the elderly.

While most evidence points to the importance of extrinsic factors, there are alternative explanations of microglia senescence and malfunction in the aged brain. It has been suggested that microglia do not constitute a single uniform cell population, but rather comprise a family of cells with diverse phenotypes. Once activated, they are committed to various phenotypes[38,40,69]. Whether it is possible to avoid the commitment to a destructive phenotype or at least to change the commitment, if the activation has already occurred, remains unknown. There is also evidence implicating intrinsic alterations as the cause of microglia senescence[70], which is actually easier to accept since aging is generally considered to be a natural course initiated by gene alterations. The exact cause of microglia senescence and malfunction in the aged brain clearly need further investigation, as it might provide key insight into the mechanistic switch between neuroprotective function and neurotoxicity of the cells.

## Molecular basis of microglia dysfunction in the aged brain

Little is known about the molecular basis of microglia dysfunction in the aged brain. It may closely correlate to and share the similar molecular mechanisms of general aging, which is an extremely complex multifactorial process including molecular, cellular, and systemic levels and even the interactions among these three levels. Many genes change expression with age. DNA microarrays have been used to determine genome-wide transcriptional changes with age and allow researchers to compile a transcriptional fingerprint of "normal" aging and to compare it with that of neurodegeneration. This approach enables the identification of gene expression changes relevant to senescence and neurodegeneration. In a study that screened 13,500 *Drosophila *genes in young, old, and oxygen-stressed flies, Landis et al. found upregulation of genes related to purine biosynthesis, heat-shock proteins, antioxidants, and innate immune responses in old flies and in oxygen-stressed flies, suggesting the specific gene expression profile during aging[71]. Insulin-like signaling pathway has been shown to regulate life span in worms, flies, and mice[72].

In the mouse hippocampus, the basal expression of 128 genes changes with aging[73]. The genes whose expression is increased in the aged mouse hippocampus include the MHC TL region, thymic shared antigen (TSA-1), cytokine (IL-1β), TNF-α, and chemokine (MCP-1), suggesting that expression of the immune-related genes changes with aging in the brain. In an established mouse model of AD (PS1-APP mice), altered expression of many genes was seen in microglia from old mice, but not from younger ones, when compared with their littermate controls. These genes include Aβ-binding scavenger receptors A (SRA), CD36, receptor for advanced glycosylation end products (RAGE), Aβ-degrading enzymes (insulysin, neprilysin) and MMP9[74]. An altered gene expression profile has also been seen in microglia from AD brain. Walker et al[70] demonstrated that when compared with those from non-demented brains, large alterations in gene transcription were found in microglia derived from postmortem AD brain, including upregulation of many proinflammatory cytokines and chemokines such as IL-1β, IL-8, and matrix metalloproteinases (MMP). Deregulation of these genes might underlie the mechanisms of microglia dysfunction in the aged brain and in neurodegeneration.

## Role of microglia in Parkinson's disease and Alzheimer's disease

**Figure 1:**
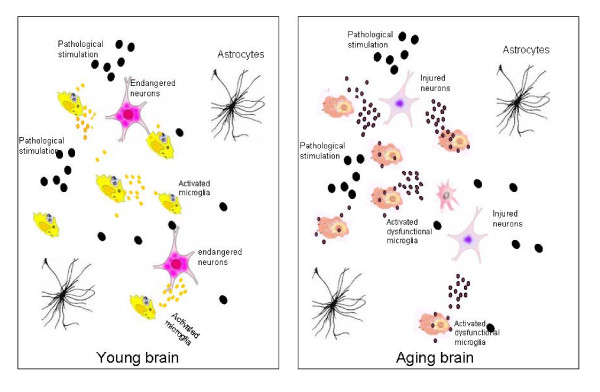
**Age-primed microglia hypothesis of Parkinson's disease**. Microglia functions differentially in the substantia nigra of the young (left) and aged (right) brain. **Left**: When facing pathogenic stimuli (large black dots), the healthy microglia in the young brain respond by releasing neurotrophic factors (small yellow dots) to support the endangered dopaminergic neurons and limit neuronal damages. **Right: **In the aged brain the microglia are primed with aging and function abnormally. When exposed to pathogenic stimuli, they are overactivated and release excessive oxidative stress and inflammatory factors (small black dots), which damage the vulnerable dopaminergic neurons and eventually lead to neurodegeneration.

The considerable contribution of microglia activation to the pathogenesis of PD has long been proposed[45,46,75]. Aging is the strongest risk factor for PD, which is true not only in the epidemiological studies, but also in the models of PD. A low systemic dose of rotenone had no effect on young rats, but led to a 20-30% reduction of dopaminergic neurons in the substantia nigra of older rats[76]. Similar phenomena replicate in MPTP-induced dopaminergic neuronal loss in elderly mice, in which more severe and persistent microglia activation associated with TH neuronal loss was found in MPTP-treated elderly rodents[77], suggesting that age-associated microglia over-activation may contribute to the increased sensitivity of dopaminergic neurons to neurotoxins. A recent study demonstrated that whether microglia are neuroprotective or neurotoxic to dopaminergic neurons is age-dependent[48], in which activated microglia switches from neuroprotective in the neonatal brain to neurotoxic in the aged brain. It appears that aging could be the determining factor of microglial functions. In other words, microglia senescence that occurs in the aged brain is responsible for the functional alterations and dysregulated responses of microglia. In the aged human brain, a significantly greater area of the substantia nigra is occupied by microglial cell bodies and processes than that of younger subjects[78]. Moreover, the dopaminergic neurons are more sensitive to oxidative stress imposed by microglia than other types of neurons[79]. Thus, increased microglia activation[78] and/or senescenced microglia in the aged brain has put the dopaminergic neurons in a more dangerous environment than that of the young brain. We, therefore, propose that in PD microglial function changes with age by releasing more inflammatory cytokines or oxidative stress, which prime microglia in the substantia nigra to be in an activating state and cause a dysregulated microglia response to pathogenic stimuli from the environment. Under this scenario, previously neurotrophic microglia turn into neurotoxic and release less-controlled oxidative products and overproduce inflammatory cytokines, which eventually promotes or leads to dopaminergic neurodegeneration (Figure 1). The similar scenario may also occur in AD, another notable age-related neurodegenerative disease, in which microglia has also been considered to be a major player in its pathogenesis. Microglia over-activation and dysfunction are seen in AD brain. Clustering of microglia are usually found around the senile plaques in the aged non-demented and AD brain[22]. Aging microglia are more prevalent in AD brain than age-matched, non-demented brain[54]. Active microglial cells are needed as scavenger cells in the CNS. However, both compromised amyloid-clearing ability of microglia[80] and the damage of microglia by amyloid[81] have been reported in AD brain. By not responding to normal regulatory feedback mechanisms and/or having an impairment in their ability to clear Aβ, microglia cells lose their ability to handle potentially toxic compounds and become cytotoxic due to their persistent activation[77]. Thus, Bernhardi et al. proposed that AD is not caused by hyperactive but rather by dysfunctional microglia[82]. In a recent study, Streit et al. found dystrophic (fragmented) rather than activated microglial cells are colocalized invariably with degenerating neuronal structures positive for tau (neuropil threads, neurofibrillary tangles, neuritic plaques) in the brains with AD pathologies[83]. The microglial dystrophy even precedes the spread of tau pathology. these observations support the idea that microglia senescence rather than microglia activation contributes to the pathogenesis of AD. Further investigation of microglia aging or dysfunction in AD may offer a unique approach in probing the microglia biology and thus a new therapeutic direction in preventing and treating AD.

## Conclusion remarks

Aging is an extremely complex, multifactorial process with deregulation of the immune system. A wide spectrum of changes occurs in both adaptive and innate immune systems, particularly in the myeloid lineage, including the microglia, in the aged brain. Many neurodegenerative diseases are age-related, and the neuroinflammation characteristic of chronic reactive microgliosis is thought to contribute to the age-related neurodegeneration. Due to the original sentinel role and being essential cells of the brain, microglia can hardly evolve to threaten the neuron. Much evidence has emerged to suggest the neuroprotective effect of microglia under specific circumstances. The traditional view of the detrimental activated microglia needs to be reappraised. An alternative new hypothesis of microglia senescence has been proposed, which gives novel viewpoints on this issue. Whether microglia dysfunction is determined by their intrinsic changes or results from a response to altered environment in the aging process remains controversial. Whether microglia activation is a curse or blessing appears to be dependent on the timing, the environment, and the nature of the stimuli. More studies are needed for further understanding of the detailed regulation of microglia activation and microglia senescence during aging and the mechanism of microglia over-activation under specific neurodegenerative conditions. Revealing the molecular mechanisms of microglia dysfunction in neurodegeneration will help develop therapeutic strategies for treating aging-related neurodegenerative diseases.

## Competing interests

The authors declare that they have no competing interests.

## Authors' contributions

X-GL, J-QD and S-DC wrote the manuscript. All authors read and approved the final manuscript.
